# Hierarchical pulmonary target nanoparticles *via* inhaled administration for anticancer drug delivery

**DOI:** 10.1080/10717544.2017.1365395

**Published:** 2017-08-28

**Authors:** Rui Chen, Liu Xu, Qin Fan, Man Li, Jingjing Wang, Li Wu, Weidong Li, Jinao Duan, Zhipeng Chen

**Affiliations:** a College of Pharmacy, Nanjing University of Chinese Medicine, Nanjing, China;; b Collaborative Innovation Center of Chinese Medicinal Resources Industrialization, Nanjing, China

**Keywords:** Inhaled administration, hierarchical target, mitochondria, drug delivery, pulmonary anticancer drug

## Abstract

Inhalation administration, compared with intravenous administration, significantly enhances chemotherapeutic drug exposure to the lung tissue and may increase the therapeutic effect for pulmonary anticancer. However, further identification of cancer cells after lung deposition of inhaled drugs is necessary to avoid side effects on normal lung tissue and to maximize drug efficacy. Moreover, as the action site of the major drug was intracellular organelles, drug target to the specific organelle is the final key for accurate drug delivery. Here, we designed a novel multifunctional nanoparticles (MNPs) for pulmonary antitumor and the material was well-designed for hierarchical target involved lung tissue target, cancer cell target, and mitochondrial target. The biodistribution *in vivo* determined by UHPLC–MS/MS method was employed to verify the drug concentration overwhelmingly increasing in lung tissue through inhaled administration compared with intravenous administration. Cellular uptake assay using A549 cells proved the efficient receptor-mediated cell endocytosis. Confocal laser scanning microscopy observation showed the location of MNPs in cells was mitochondria. All results confirmed the intelligent material can progressively play hierarchical target functions, which could induce more cell apoptosis related to mitochondrial damage. It provides a smart and efficient nanocarrier platform for hierarchical targeting of pulmonary anticancer drug. So far, this kind of material for pulmonary mitochondrial-target has not been seen in other reports.

## Introduction

Among all cancers, primary lung cancer is one of the leading causes of cancer-related death, and the lung is also a major site of metastasis for other cancers. Due to the poor efficacy of intravenous chemotherapy against lung tumors and little drug access to lung tissue after intravenous administration, the lung-resident cancers are difficult to treat *via* conventional intravenous chemotherapy, and mortality rates are high (Carvalho et al., 2014). Compared with intravenous administration, direct inhaled administration of chemotherapeutic drugs to the lungs significantly enhances drug exposure to lung-resident cancer cells and may improve chemotherapy. Thus, inhaled administration becomes a priority in terms of curing a wide variety of lung-related diseases, including lung cancer (De et al., 2015; Goyal et al., 2015; Tewes et al., 2015; Rahhal et al., 2016).

However, direct inhalation of cytotoxic drugs may cause respiratory system damage and high concentrations of acute poisoning at the lung tissue (Chiang et al., 2010; Nørgaard et al., 2010). More importantly, even if most of the inhaled drug successfully lands in the lung area, drug concentration in a lung-resident tumor or cancerous cell may still be below requirements, due to lack of tumor targeting. Thus, a number of nanoparticle and liposomal formulations based on nanotechnology have been evaluated as delivery systems to realize cellular level targeting (Garbuzenko, 2011; Alfagih et al., 2015). Park used an affinity molecule to bind to lung epithelium which may prolong retention of therapeutic molecules within the lung and consequently yield higher overall bioavailability (Park et al., 2014). Kusumoto developed a multifunctional envelope-type nanodevice that targets the lung endothelium, delivers its encapsulated siRNA to the cytoplasm, and eradicates lung metastasis (Kusumoto et al., 2013). Kaminskas proved that PEGylated nanostructure have potential as inhalable drug delivery systems to promote the prolonged exposure of chemotherapeutic drugs to lung-resident tumors and to improve antitumor activity (Kaminska et al., 2014). These studies provide promising indications for inhaled pulmonary treatment with reduced lung-related side effects and improved antitumor activity. Therefore, further cancerous cellular targeting should be considered for nanocarriers after lung tissue target by inhaled administration.

As many pharmacological intervention points were the intracellular organelle, drug delivery aimed at specific organelle target became a hot research issue recently (Dong et al., 2011; Wang et al., 2011; Biswas et al., 2012). The mitochondrion was a prime target for pharmacological intervention, which play vital functions in the cell's energy metabolism and the regulation of programed cell death. It controls the activation of apoptotic effector mechanisms by regulating the translocation of pro-apoptotic proteins from the mitochondrial inter-membrane space to the cytosol. Similarly, other signs of cancer, such as unlimited proliferation, nonsensitive to the growth signal, impaired apoptosis, increased metabolism, and decreased autophagy were related to the damage of mitochondria (Modica-Napolitano & Singh, 2002). Based on the vital role of mitochondria in the occurrence, development, and death of cancer cells, making the mitochondria become one of the most attractive drug target sites (Malty et al., 2014). Therefore, in order to further improve the drug efficacy, it is necessary to design mitochondrial targeted compounds for intracellular organelle target after cancerous cellular target.

Yuanhuacine is one natural structure extracted from the Chinese medicine *Thymelaeaceae*, which has been used for the treatment of ascites, cough, and asthma in modern clinic. Recently, yuanhuacine is also reported to act as an antitumor component for cancer treatment, especially effective for lung cancer (Hong et al., 2010). Compared from the *in vitro* inhibitory activity of yuanhuacine against P-388 lymphocytic leukemia, A549 human lung cancer cells, and HMEC endothelial cell, it exhibits severely significant inhibitory activity against lung cancer cells (A549) (Zhan et al., 2005). It has been found that yuanhuacine can activate cell apoptotic process, though its inhibitory activity against HL-60 human promyelocytic leukemia cells and SNU-1 gastric cancer (Park et al., 2007). The anticancer mechanism of yuanhuacine has also been investigated by Zhang et al. (2006). However, yuanhuacine has server system toxicity, especially evident for liver, kidney, and reproductive function through systemic administration such as oral and intravenous delivery (Chen et al., 2013; Jiang et al., 2014). Therefore, to reduce toxicity and increase efficiency of yuanhuacine in lung cancer treatment, the biggest obstacles is how to concentrate yuanhuacine in lung area and decrease the amount in other organs (especially in reproductive) (Zhang et al., 2009).

**Figure 1.**
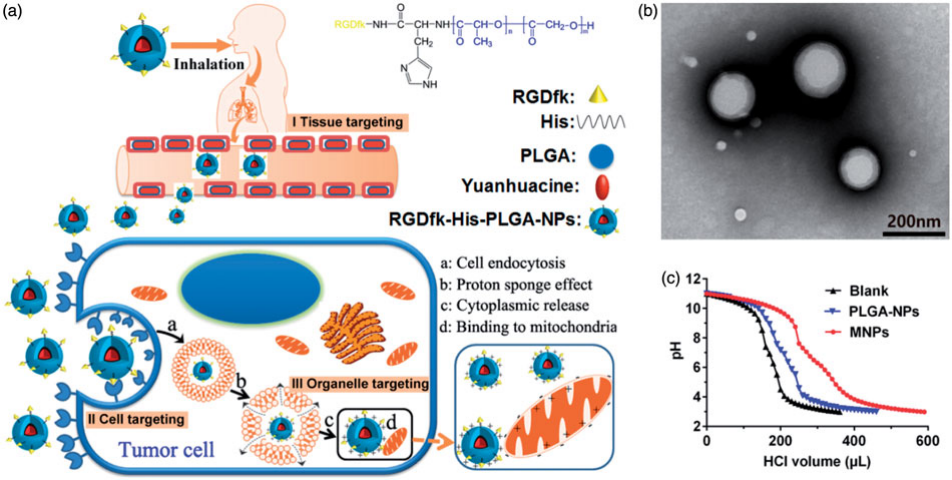
(a) Schematic fabrication of intelligent RGDfk–histidine–PLGA-NPs for pulmonary mitochondrial-targeted drug delivery. (b) TEM images of yuanhuacine/MNPs. (c) Acid titration profiles of blank solution, PLGA-NPs, and MNPs solutions.

In our present study, yuanhuacine is applied to be encapsulated in a new developed nanocarrier for accurate pulmonary drug delivery using the hierarchical target strategy (Chen et al., 2015). The compound of RGDfk–histidine–PLGA was well-designed with the specific structure to realize the hierarchical target function which is illustrated in Figure 1(a). Firstly, inhalation administration is chosen to deliver the drug to deeper lung tissue. The biocompatible and biodegradable materials approved by FDA, poly (lactic-*co*-glycolic acid) copolymers (PLGA), were chosen as prime inhaled material, with a percentage of the lactose mixture as carrier material. Through regulating the size of carrier material and the affinity between nanoparticles and carrier material, carrier material could deliver most nanoparticles to deep lung area though inhaled administration, which make prime tissue target (Stage I). Then, to deliver drug into the lung-resident cancer cells, RGDfk receptor was bond on the surface of the NPs *via* chemical grafting to recognize the integrin α_v_β_3_ receptor on the lung cancer cell membrane, and the NPs could concentrate on the cell surface and internalization to achieve cancer cellular target (Stage II). Finally, to achieve the intracellular lysosome escape and mitochondrial target, histidine groups with highly positive charge were engineered into the NPs to make endosomes proton influx and bursting (called ‘proton sponge’ effect), and facilitate the positive NPs close with negative mitochondrial membrane, which make precise drug delivery to organelle (Stage III). These intelligent nanocarriers can realize multistage target successively and accurate drug delivery to mitochondria for pulmonary tumor treatment.

## Methods

### Materials

Histidine benzyl ester *p*-toluenesulfonate was received from Hanhong Chemical Co., Ltd. (Shanghai, China). Carboxyl terminated poly lactic acid ethanol copolymer OH-PLGA-COOH75/25 (*M_W_*: 15000) was obtained from Dai Gang Biological Engineering Co. Ltd. (Ji'nan, China). RGDfk was purchased from Aite Biotechnology Co. Ltd. (Nanjing, China). Sodium bicarbonate, ethyl acetate, anhydrous sodium sulfate, methanol, dichloromethane (DCM), *N*,*N*-dimethylformamide (DMF), hydrazine hydrate, formic acid, and dichloromethane were purchased from Nanjing Chemical Reagent Co., Ltd. (China). *N*,*N*′-dicyclohexyl carbodiimide (DCC), 1-hydroxybenzotriazole (HoBt), 4-dimethylaminopyridine (DMAP), *N*-hydroxysuccinimide (NHS), Palladium on carbon and ninhydrin were purchased from Aladdin Biochemical Technology Co., Ltd. (Shanghai, China). Yuanhuacine, raw material medicine (>98% purity), was purchased from the standardization of traditional Chinese medicine research center (Shanghai, China). Polyvinyl alcohol (PVA) was purchased from Aladdin Biochemical Technology Co., Ltd. (Shanghai, China). Lactose was purchased from Germany. Clarithromycin with purity of 98% or higher was purchased from Tokyo Chemical Industry Co., Ltd. (Tokyo, Japan). Ether was purchased from Shanghai Ling-feng Chemical Reagents Company (Shanghai, China). RPMI medium1640 basic was from GIBCO (Grand Island, NY). All other materials and reagents used in this study were of analytical grade and were used without any further modification.

Human lung cancer cell line A549 was purchased from the Cell Bank of Type Culture Collection of the Chinese Academy of Sciences (Shanghai, China). Fifty-two male Sprague-Dawley rats, weighed 180–220 g, were supplied by the Experimental Animal Center of Nanjing University of Chinese Medicine. The rats were kept in an environmentally controlled breeding room for 5 days at a temperature of 22–25 °C and a relative humidity of 50 ± 10% with food and water provided *ad libitum*.

### Synthesis of RGDfk–histidine–PLGA

Histidine benzyl ester *p*-toluenesulfonate (2 g, 3.4 mmol) was dissolved in water at room temperature and adjusted to pH 8.5 with sodium bicarbonate. Then two times volume of ethyl acetate was added to the solution for extraction and repeated twice, then combined ethyl acetate layer successively. The mixture was dehydrated with hydrous sodium sulfate, then filtered, and filter liquor was evaporated with vacuum distillation at 37 °C for 60 min to obtain histidine benzyl ester. PLGA (1 g, 0.667 mmol) was dissolved in 10 mL DCM added with 2 mL of DMF with DCC (0.1 g, 0.485 mmol) and HoBt (0.1 g, 0.740 mmol) were was added to the PLGA solution successively, and then triethylamine (0.2 mL) was added to the solution under magnetic stirring for 5 h. The histidine benzyl ester (0.1 g) was dissolved in appropriate volume of DCM and then added to the solution and the reaction was stirred overnight at room temperature. After the reaction time, the solid impurities were removed by filtration, then the filtrate was transferred to a dialysis bag (*M_W_*: 8000–14,000) with the mixed solvent of DCM and DMF as dialysis medium, and left overnight under magnetic stirring. Then the DCM was evaporated with vacuum distillation and the ice water was added successively, and then the product was filtered followed by freeze-drying to obtain histidine benzyl ester–PLGA. Histidine benzyl ester–PLGA was dissolved in DCM with palladium on carbon as a catalyst and then the reaction was carried out under a hydrogen atmosphere and stirred for 48 h at room temperature. After the reaction time, diatomite was added to the reaction solution, then the reaction mixture was filtered, and then the filtrate was transferred to a dialysis bag (*M_W_*: 8000–14000) with DCM as dialysis medium and left overnight under magnetic stirring. Finally, the solution was evaporated with vacuum distillation to obtain the histidine–PLGA. Histidine–PLGA (0.225 g, 0.015 mmol) and NHS (0.026 g, 0.226 mmol) were dissolved in a proper amount of DMF. DMAP (0.055 g, 0.450 mmol) and DCC (0.046 g, 0.223 mmol) were dissolved in DMF, then it was added dropwise into the solution and stirred for 2 h at room temperature. Then RGDfk (0.011 g, 0.018 mmol) was added to the reaction solution and then stirred for 24 h. After the reaction time, solid impurities were removed by filtration, then the filtrate was transferred to a dialysis bag (*M_W_*: 8000–14000) with DMF as dialysis medium. Then the product was washed with water and extracted with DCM; subsequently the DCM layer was evaporated with vacuum distillation to obtain the target product, RGDfk–histidine–PLGA.

### Preparation of RGDfk–histidine–PLGA multifunctional nanoparticles (MNPs)

Nanoparticles were prepared by the emulsion solvent evaporation method. Specifically, RGDfk–histidine–PLGA (20 mg) was dissolved in a mixed organic solvent consisted of DCM and methanol (400 μL, *v*:*v* = 9:1) . The solution above was added to 2% PVA (3 mL), and 5 mL distilled water was added successively, then stirred over night at room temperature to remove DCM and MNPs were obtained. Drug-loaded nanoparticles, named yuanhuacine/MNPs, were prepared as above by adding yuanhuacine (1 mg) in the mixed organic solvent. PLGA-NPs were also prepared as above, using PLGA instead of RGDfk–histidine–PLGA.

### Characterization of nanoparticles

The particles size and size distribution were measured using Malvern Zetasizer Nano ZS90 instrument (Malvern Instruments Ltd., UK) at a concentration of 40 mg/mL. All measurements were carried out at room temperature and the data were achieved with the average of three measurements. The morphology of the nanoparticles was examined using a transmission electron microscope (TEM, Hitachi, Japan). The nanoparticle suspension was dropped on the copper mesh coated with a support film and then stained with phosphotungstic acid stain (2%) for 1 min. Subsequently, the sample was dried naturally for observation. HPLC was used for the quantitative determination of yuanhuacine. For the detection of yuanhuacine, the mobile phase consisted of methanol:distilled water 82:18 (v/v) introduced at a flow rate of 1 mL/min and the detection wavelength was 233 nm. A Merck-C18 column (4.6 mm ×250 mm, pore size 5 mm) was used. According the peak area (*A*) and the concentration (*C*), the standard curve was simulated: *A* = 19,910*C* − 12788 (*r*^2^ = 1) and the linear range of 2.5–80 μg/mL. Ultra-high-speed centrifugation technique and HPLC were used to determine the drug encapsulation efficiency (EE) and drug loading content (LC) of yuanhuacine/MNPs and yuanhuacine/PLGA-NPs. Specifically, 1.0 mL suspensions contained an amount of yuanhuacine/MNPs, and yuanhuacine/PLGA-NPs was placed in a 1.5 mL centrifuge tube matched with ultra-high-speed refrigerated centrifuge (Amicon ultra, Millipore Co., Billerica, MA) and was centrifuged for 30 min at 4 °C, 20,000 rpm. The unencapsulated yuanhuacine was determined by HPLC as described above. The EE and LC were calculated by the following equations: EE (%) = (*M* − *M*_1_)/*M* × 100%, LC (%) = (*M* − *M*_1_)/*W* × 100%, where *M*_1_ is the amount of yuanhuacine in supernatant, *M* is the total amount of yuanhuacine, and *W* is the quality of the freeze-dried nanoparticles after removing the free yuanhuacine.

### Determination of the buffering capacity and *in vitro* evaluation of drug release

The buffering capacity of MNPs and PLGA-NPs was determined by acid–base titration over a pH range from 11.0 to 3.0 according to our previous study (Chen et al., 2015). Briefly, solutions of each sample were adjusted to pH 11 using 0.1 mol/L NaOH and were then titrated to pH 3 with 0.05 mol/L HCl .

Both of the release studies of yuanhuacine/MNPs and yuanhuacine/PLGA-NPs were carried out in phosphate buffer solution (PBS, 500 mL, pH 7.4) with 1% Tween 80 at 37 °C and the stirring speed was 50 r/min. One milliliter of yuanhuacine/MNPs and yuanhuacine/PLGA-NPs were placed in the dialysis bag (*M_W_*: 8000–14,000) with concentration of 46.6 and 48.8 mg/mL respectively, corresponding to 2 mg/mL of yuanhuacine. *In vitro* release testing protocols were performed and monitored by HPLC. 1 mL of the solution was collected for analysis and this volume of fresh PBS was returned to the release medium. The release amount of yuanhuacine with time was recorded and the release profile was calculated and drawn.

### Preparing of dry powder for pulmonary inhalation

Dry powder for pulmonary inhalation was first prepared though mixing yuanhuacine/MNPs with lactose. Different prescriptions of rough lactose, fine lactose were weighted and mixed with yuanhuacine/MNPs, to obtain different size of dry powder for inhalation. Powder Compacting NGI 170 (MSP Corporation, Shoreview, MN) had a simulation of drug delivery to lung in 60 L/min flow, with methanol/water (*V*:*V* = 1:1) rinse all the tray and the adapter component. After it was centrifuged at 12,000*g* for 5 min, the supernatant was used for the determination of yuanhuacine. The simulation of lung deposition *in vitro* is to determine the suitable size of dry powder for following study.

### UHPLC–MS/MS analysis to quantify yuanhuacine *in vivo*

A DGU-20A 5R series UHPLC system equipped with a LC-10ATvp binary pump (SHIMADZU, Japan) was used for UHPLC–MS/MS analysis. A 5500 triple quad tandem mass spectrometer equipped with electrospray ionization source (AB Sciex, Concord, OH, Canada) was used for mass spectrometry. The mobile phase was composed of a mixture of 0.1% formic acid aqueous solution (A) and acetonitrile (B) with a gradient elution program (0–0.1 min, 40% B; 0.1–2.5 min, 40–95% B; 2.5–3.5 min, 95% B; 3.5–4.5 min, 95–40% B; 4.5–5.0 min, 40% B). The flow rate was set at 0.3 mL/min and the injection volume was 5 µL. The ESI source was operated in positive ionization mode. The mass spectrometer was operated in multiple reactions monitoring (MRM) mode and the selected monitor ions were *m/z* 649.4/151.1 for yuanhuacine and *m/z* 748.5/590.4 for IS. The optimized parameters were as follows: ion source temperature (TEM), 550 °C; curtain gas (CUR), 35 psi; ion source gas 1 (GAS1), 55 psi; ion source gas 2 (GAS2), 55 psi; ion spray voltage (IS), 5500 V.

### Pharmacokinetics

Twelve male Sprague-Dawley rats were randomly divided into two groups (*n* = 6) for intravenous and inhaled administration of yuanhuacine/MNPs, respectively. DP-4M pulmonary drug delivery device (Penn-Centurythe Company, Philadelphia, PA) was used for the inhaled administration. Six rats each was received the same dose of yuanhuacine (100 µg/kg). Before testing the rats were fasted overnight with free access to water. All animal experiments were carried out according to the Guidelines for the Care and Use of Laboratory Animals. For intravenous groups, blood samples were collected with heparinized microfuge tube at 0.5, 1, 1.5, 2, 4, 6, 8, 10, 12 h post-dosing. For inhaled groups, blood samples were collected at 2, 4, 6, 8, 10, 12, 20, 40, 60, 80, 100, 120 h post-dosing. Then blood samples were centrifuged at approximately 12,000 rpm for 5 min to obtain plasma. After collection, samples were stored at −20 °C until UHPLC–MS/MS analysis. To 100 µL plasma sample, 10 µL of IS solution and 400 µL of Na_2_CO_3_ solution (0.02 M) were added into an eppendorf tube. The mixture was spiked with 1 mL extraction solvent (ether:dichloromethane =8:1) and then eddied for 5 min of extraction. After centrifuged at 12,000 rpm for 5 min, the supernatant was then transferred into another eppendorf tube and blown to dryness with nitrogen at 37 °C. The residue was reconstituted into 100 µL methanol, and centrifuged (12,000 rpm for 10 min). The supernatant of 5 µL was injected into UHPLC–MS/MS system for quantitative yunhuacine based on an established analysis method analysis (Li et al., 2016).

### Biodistribution

Forty rats were randomly divided into two groups (*n* = 4) for intravenous and inhaled administration of yuanhuacine/MNPs with each rat at a yuanhuacine dose of 100 μg/kg. The rats were sacrificed at different time (1, 2, 4, 6, 9 h) after drug administration, and different tissues were collected and weighed, including liver, spleen, kidney, lung, and reproductive. The tissues were stored at −20 °C until UHPLC–MS/MS analysis. The amounts of yuanhuacine in different tissues were calculated as the amount of yuanhuacine (ng)/the weight of the tissues (g), respectively.

### Cellular uptake and endocytosis pathways

A549 cells were seeded in 12-well plates at a density of 8 × 10^4^ cells per well. After incubating for 24 h at 37 °C, the culture medium was replaced with RPMI 1640 medium containing drugs for co-incubating 2 h. Then culture medium was removed followed by washing with PBS for three times at 4 °C. Trypsin was added to for digestion, then complete medium was added to stop digestion and the cells were collected. After centrifuging for 5 min at 1000 rpm the supernatant was discarded, then the cells were suspended in 1 mL PBS successively, centrifuged for 5 min at 1000 rpm and then the supernatant was removed. A 100 μL of RIPA lysate was added for Lysis of cells, then mixed gently with pipetting, and then centrifuged for 5 min at 12,000 rpm. The supernatant was collected and labeled as supernatant I, then 200 μL of methanol was added to 50 μL of supernatant I successively, vortexed for 5 min, and then centrifuged at 12,000 rpm for 10 min. Finally, the supernatant of 5 µL was injected into HPLC–MS/MS system for analysis. The concentrations of yuanhuacine in the supernatant were calculated using the standard curve: *A* = 3826.0925*c* − 11920.50554 (*r*^2^ = 0.9994, linear range: 50–4000 ng/mL). To analyze the protein content in cells, 20 μL of supernatant I was taken and then protein concentration was determined using Enhanced BCA Protein Assay Kit (Beyotime). According the absorbance (*A*) and the protein concentration (*c*) the standard curve was simulated: *A* = 0.6626 × *c* + 0.0529 (*r*^2^ = 0.9990) and the linearity was good in the range of 0.025–0.5 mg/mL. Uptake of yuanhuacine was calculated as the amount of yuanhuacine (mg)/the amount of cells protein (mg), respectively.

To explore the factors on cellular uptake, different concentrations (0. 25, 1, 2.5, 5, 12 μM), co-incubated time (10, 30, 60, 90, 120, 180, 240 min), and temperature (4 and 37 °C) were investigated. To understand the cellular internalization pathways, different kinds of endocytosis inhibitor, such as chlorpromazine (20 mg/mL), nystatin (10 μg/mL), amiloride (50 μM), and sodium azide (1 mg/mL), were added into the cells for 1 h before drugs were added for another 2 h. Then HPLC–MS/MS assays were conducted to determine yuanhuacine. Compared with the control cells without inhibitors, the reduced uptake percentage with inhibitors indicated the corresponding endocytosis pathways.

### Confocal laser scanning microscopy (CLSM) observation

To verify the endolysosomal escape and mitochondrial targeting function of MNPs, the double-labeling experiments using CLSM was carried out on A549 cells. The NPs were labeled by C6 dye, while lysosomes and mitochondria were labeled by LysoTracker Red (Invitrogen, Carlsbad, CA) and MitoTracker Red (Invitrogen), respectively. The cells were seeded at a density of 1 × 10^5^ cells per well for 24 h at 37 °C, and added with 200 ng/mL of C6/PLGA NPs or C6/MNPs for another 2 h. Then 50 nM LysoTracker and 200 nM MitoTracker were added, respectively, to stain cells for 30 min at 37 °C. The cells were washed by 4 °C PBS thrice, fixed and observed under CLSM (Olympus, Japan).

### Cell toxicity assay

MTT assay were used to assess the *in vitro* cytotoxicity of yuanhuacine, yuanhuacine/PLGA-NPs, and yuanhuacine/MNPs. A549 cells were seeded in 96-well plates at a density of 2 × 10^4^ cells per well with RPMI 1640 medium containing 10% FBS for 24 h. Then the medium was replaced with 100 μL fresh medium containing different concentrations of yuanhuacine, yuanhuacine/PLGA-NPs, and yuanhuacine/MNPs, respectively, and incubated for 24 h at 37 °C. PBS containing 5 mg/mL MTT was added to the cells (20 μL/well). After incubation for 4 h, the supernatant was removed and DMSO was added to the cells (150 μL/well). The resulting solution was measured the absorbance at 490 nm using a microplate reader (Thermo Scientific, Waltham, MA). The data were expressed as the inhibitory rates of the treated cells to the untreated cells.

### Cell apoptosis and cell cycle detect

#### Annexin V-FITC/PI stain to detect cell apoptosis

A549 cells were seeded in 6-well plates at a density of 2.4 × 10^5^ cells per well for 24 h at 37 °C. Then the cells were treated with yuanhuacine, yuanhuacine/PLGA-NPs, and yuanhuacine/MNPs at 40 μg/mL per well, respectively, with the cells without treatment as control. After incubation for 24 h, the cells were washed by PBS twice, trypsinized, centrifuged, and collected at 1000 rpm for 5 min, then washed by PBS. The cells were resuspended into 100 μL binding buffer, and added with 5 μL Annexin V-FITC and 5 μL PI staining solution. After the reaction for 10 min in dark, another 400 μL binding buffer was added and mixed. Then the samples were analyzed using a flow cytometer system (Cytomics™ FC500 Flow Cytometer, BECKMAN COULTER, California).

#### Cell cycle analysis

A549 cells were seeded and treated with different formulations, washed, trypsinized, and centrifuged as above. Then cells were fixed with cold 70% ethanol for 24 h at 4 °C, and centrifuged again at 1000 rpm for 5 min, then washed by PBS. The cells were resuspended into 500 μL PI staining solution. After reaction in dark for 30 min, the samples were analyzed using the flow cytometer system.

#### Cell nucleus stain

A549 cells were seeded in 96-well plates at a density of 1.5 × 10^4^ cells per well for 24 h at 37 °C, and treated with different formulations. After incubation for 24 h, the cells were washed by PBS twice and 100 μL paraformaldehyde was added to fix cells for 10 min. The cells were washed by PBS twice and 100 μL hoechst 33258 stain solution was added for 5 min. After washed by PBS twice, the cells were observed under a microscope.

### Cell apoptosis mechanism analysis

#### Determine of mitochondrial membrane potential (△Ψm)

A549 cells were seeded and treated with different formulations, washed, trypsinized, and centrifuged as above. The cells were resuspended into 500 μL and added 1 mL JC-1 dye stain solution incubated for 20 min. During the incubation, JC-1 buffer was diluted by distilled water according to the ratio of 1:4 (V/V). The cells were centrifuged at 600 rpm for 4 min and washed twice by JC-1 buffer. Then the cells were resuspended by moderate JC-1 buffer and assayed using the flow cytometer system.

#### Determine of Cyt C expressions

A549 cells were seeded and treated with different formulations, washed, trypsinized, and centrifuged as above. A 0.5 mL paraformaldehyde was added to fix cells and paraffin section was prepared. The section was stained using a standard immunohistochemistry method and taken photographs to examine Cyt *C* expressions.

## Results and discussion

### Synthesis and characterization of RGDfk–histidine–PLGA

The synthesis of RGDfk–histidine–PLGA included four steps chemical reaction as shown in Figure S1. Briefly, histidine benzyl ester was first obtained by extraction, then it was grafted to PLGA by amide condensation reaction between amino group on histidine benzyl ester and carboxyl groups on PLGA. Subsequently, the histidine benzyl ester–PLGA was hydrogenated to obtain histidine–PLGA. Finally, RGDfk–histidine–PLGA was synthesis by amide condensation reaction between histidine–PLGA and RGDfk. The product was purified by dialysis and stored in 4 °C after freeze-drying. The structure of every product was confirmed through ^1^H NMR spectra in Figures S2–S5.

### Preparation and characterization of RGDfk–histidine–PLGA NPs

Multifunctional RGDfk–histidine–PLGA NPs (MNPs) were prepared *via* solvent evaporation method as mentioned above. The particle sizes and size distribution of yuanhuacine encapsulated MNPs were measured using dynamic light scattering (DLS) and the mean sizes of yuanhuacine/MNPs were 153.4 ± 6.9 nm. The morphology study by TEM images has shown that the yuanhuacine/MNPs had a nearly-spherical shape and the size was a little smaller than the results of DLS because of dry state (Figure 1(b)). The zeta potential of MNPs solution was investigated and found it pH dependent (Figure S6). The MNPs were negatively charged in pH 7 due to the PLGA matrix which was always negatively charged, and changed to positively charge when pH decreased due to the grafted histidine groups. The isoelectric point of histidine was 7.58, and became positive below pH 7. This kind of charge reversal can be used to explain ‘proton sponge’ effect in lysosomal (pH 4.5–5.5) mentioned below.

For yuanhuacine encapsulated MNPs, the drug encapsulation efficiency (EE) was high as 90.5 ± 2.42% and the drug loading content (LC) was 4.3 ± 0.12%, which is quite similar with yuanhuacine encapsulated PLGA (the data did not shown). The *in vitro* release curves of yuanhuacine from MNPs are shown in Figure S7. The releasing rates were very fast in the initial stage, due to part of drug molecules were binding to the surface of nanoparticles which fell off fast. Later, drug release became slow, which may due to carrier degradation and the inner drug spread out to the surface of the nanoparticles. There was no significant difference in release behavior between PLGA-NPs and MNPs.

The mechanism of MNPs escape from the endosomal/lysosomal pathway after internalization was generally interpreted as ‘proton sponge’ hypothesis, which was associated to the proton influx, osmotic swelling, plasma disruption of the endosome membrane (Akinc et al., 2005). Highly positively charged cationic polymers with the large buffering capacity can play proton sponge effect to escape from endolysosomal. Here, the histidine groups of MNPs and the guanidino groups on RGDfk showed the positively charged in the acidic environment of endosomes (pH 5.5) and lysosomes (pH 4.5), which may generate endosomal/lysosomal escape action. The method commonly used to evaluate the proton sponge effect was to determine the buffering capacity of carriers from pH 11.0 to pH 3.0 (Singh et al., 2015). The buffering capacity of blank solution, PLGA-NPs, and MNPs is shown in Figure 1(d), and the calculated values of their buffer ability from basic to acidic conditions were 10.6%, 10.4%, 16.6%, respectively. It can be seen that MNPs displayed more obvious buffering effect than PLGA NPs due to the grafted histidine groups, so MNPs had the ability to play proton sponge effect and to escape from lysosomal. This result showed that the MNPs were smart and functional delivery system compared to the common PLGA-NPs.

### Lung tissue target *in vivo*

For pulmonary inhalation, dye powder was first prepared though mixing yuanhuacine/MNPs with lactose mixture. According to the supporting experiment (Figure S8), the best prescription was 6 µg/mg of yuanhuacine/MNPs added into lactose mixture with a proportion of 10:1 (rough lactose:fine lactose), and the size of dye powder should be well controlled at 1–5 μm which is suitable to prolong retention time on lung. Ryan had proved that absorption from the lung versus retention within the lung is highly size-dependent (Ryan et al., 2013). As small nanoparticles were easily aggregated and can be exclude out of body *via* exhalation, the MNPs powder must be added with rough lactose to improve flow ability. But if the drugs combined with rough lactose are too conducive to separate, it makes drug deposition in the larynx and trachea, difficult to reach deep lung. Then fine lactose was added into the powder mixture, to improve separation ability of the drug from rough lactose. The following experiments for pulmonary inhalation were all applied this prescription.

In fact, once the inhaled MNPs came into the blood from the lung capillaries, the body circulation of MNPs was similar with intravenous way (Li et al., 2009). The blood pharmacokinetics *in vivo* of yuanhuacine/MNPs was carried on rats to investigate the circulation time in the plasma. The mean concentration–time curves of the two formulation after intravenous and inhaled administration are shown in Figure S9, and the pharmacokinetic parameters (Berlin et al., 2016) calculated by DAS2.0 software (version 2.0, Mathematical Pharmacology Professional Committee of China, Shanghai, China) are shown in Table S1. It can be observed that the area under the curve (AUC_0−_*_t_*) of inhaled MNPs was larger than intravenous administration, which proved the good bioavailability of inhaled administration. The longer mean retention time (MRT_0−_*_t_*) and biological half-life (*t*_1/2_) of MNPs, as well as the later *T*_max_, consistently showed that inhaled MNPs had the more lasting circulation time than intravenous administration. The lower *C*_max_ of inhaled MNPs, compared to intravenous MNPs, indicated inhaled administration make it difficult for drug to get into the systemic circulation, which may decrease the drug toxicity of yunhuacine.

**Figure 2.**
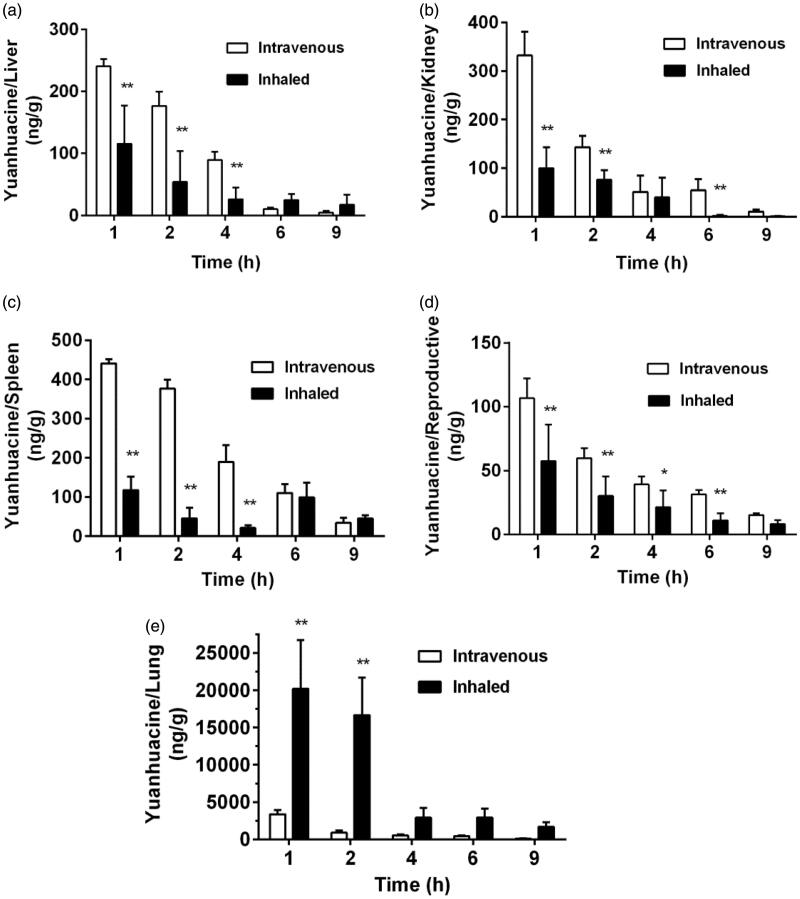
Biodistribution of intravenous or inhaled administration of yuanhuacine/MNPs accumulation in the (a) liver, (b) kidney, (c) spleen, (d) reproductive, and (e) lung at different time points with a yuanhuacine dose of 100 μg/kg (*n* = 4). *p < .05, **p < .01 versus intravenous group.

To verify the lung tissue targeting efficiency of inhaled MNPs *in vivo*, the biodistribution of two formulations including intravenous yuanhuacine/MNPs and inhaled yuanhuacine/MNPs was also carried on rats (Figure 2). It can be seen for intravenous administration, drugs highly concentrated in the reticuloendothelial system (liver, spleen, and kidney) was responsible for phagocytosis foreign substances as protective system. However, for inhaled administration, the drug concentration overwhelmingly increased in lung tissue, while the drugs in liver, kidney, and reproductive were very few and quickly eliminated. More important, as yuanhuacine had some toxicity in the reproductive system, inhaled administration can reduce its toxicity in reproductive and the resultant side effects. Targeting efficiency (Te) was defined as the relative AUC_0−_*_t_* value of target tissue to non-target tissue, which suggests the selectivity to target tissue of this drug administration. The Te value of lung towards other organs is listed in Table 1, and from the perspective of lung targeting, the inhaled administration was obviously better than intravenous injection. Therefore, inhaled administration was proved to be significant for drug deposition in lung tissue (Sullivan et al., 2015; Woods et al., 2015). At the same time, the retention time in lung of inhaled yuanhuacine/MNPs (Figure 2(e)) was quite long to maintain a high concentration. This may due to the well-controlled size (1–5 μm) of inhaled yuanhuacine/MNPs dye powder which is suitable to prolong more dose of drug retention on lung tissue.

**Table 1.** Lung targeting efficiency (Te) of yuanhuancine/MNPs.

Lung/	Intravenous	Inhaled
Liver	10.29	183.67
Spleen	4.51	116.79
Kidney	8.46	241.01
Reproductive	20.6	206.15

### Tumor cell target *in vitro*

**Figure 3.**
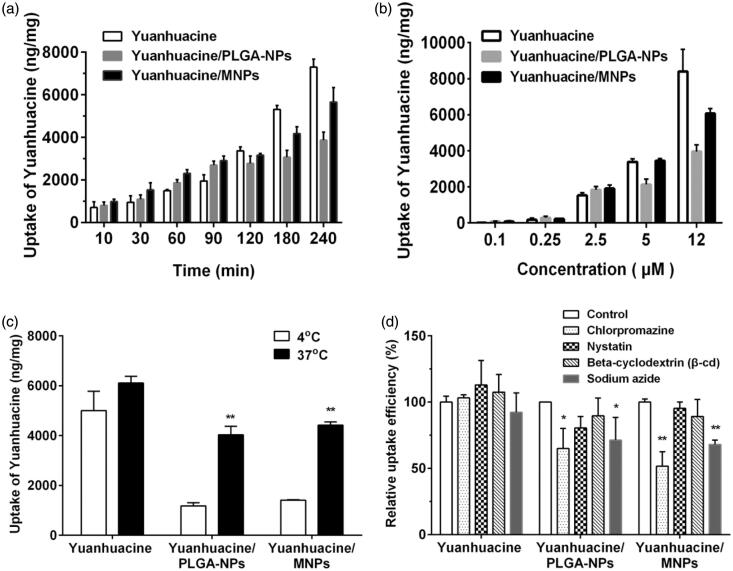
Cellular uptake on A549 cells after applying yuanhuacine, yuanhuacine/PLGA-NPs, and yuanhuacine/MNPs at (a) different time, (b) different drug concentration, (c) different temperature, and (d) relative uptake efficiency (%) in the presence of various endocytosis inhibitors. *p < .05, **p < .01 versus control.

As cellular uptake efficiency was influenced by various factors, cellular uptake assay involved different cultured time, different drug concentration, and different temperature was carried on A549 cells after applying yuanhuacine, yuanhuacine/PLGA-NPs, and yuanhuacine/MNPs. As shown in Figure 3(a,b), time- and concentration-depending increasing on cellular uptake was both displayed for each formulation. During 10–90 min, the uptake of NPs by A549 cells was higher than that of free yuanhuacie, but from 90 to 240 min, the situation is quite opposite. Similar situations have occurred on the different concentration. These results indicated cellular uptake pathway of free yuanhuacine and NPs system may be different. In general, the uptake of yuanhuacine/MNPs by A549 cells was higher than that of yuanhuacine/PLGA-NPs. When the drug concentration was 12 g/mL, the uptake of yuanhuacine/MNPs by A549 cells was about 1.54 times higher than that of yuanhuacine/PLGA-NPs. This is primarily due to the RGDkf grafted onto the surface of the MNPs which enhance receptor-mediated endocytosis.

To investigate the cellular uptake pathway, we first investigated the effect of temperature on cell uptake at 4 and 37 °C. As transport proteins have the highest activity at 37 °C and the activity was reduced when the temperature was decreased, we can determine whether transporter proteins are involved in the uptake processes through temperature change (Gabano et al., 2008). As shown in Figure 3(c), the uptake of yuanhuacine, yuanhuacine/PLGA-NPs, and yuanhuacine/MNPs by A549 cells at 37 °C was about 1.22, 3.13, and 3.42 times higher than that at 4 °C. The significant growth in cell uptake from low temperature to high proved the cellular uptake of NPs system was involved in transport proteins which may be related with energy-dependent uptake process. Conversely, yuanhuacine group, with no obvious change at 4 or 37 °C, indicated the uptake progress primarily depended on passive diffusion.

The cellular internalization mechanism has a variety of ways and the mechanism of yuanhuacine/MNPs uptake was investigated by adding different inhibitors (Figure 3(d)). Compared with other inhibitors, the presence of chlorpromazine caused the most significantly decreased on cellular uptake of yuanhuacine/PLGA-NPs (65.03%) and yuanhuacine/MNPs (51.74%) as control, indicated the cell entry pathway was mainly related to clathrin-mediated endocytosis. Then, sodium azide, an inhibitor of energy metabolism, decreased the cellular uptake of yuanhuacine/PLGA-NPs (71.34%) and yuanhuacine/MNPs (68.08%), indicated a possible energy-dependent uptake pathway, consistent with the results in Figure 3(c). Other inhibitors had no obvious impacts on relative uptake efficiency. All the results proved the cellular uptake pathway of NPs was different from free drug, and the functional MNPs may attributable to receptor-mediated endocytosis.

### Intracellular mitochondrial target *in vitro*

**Figure 4.**
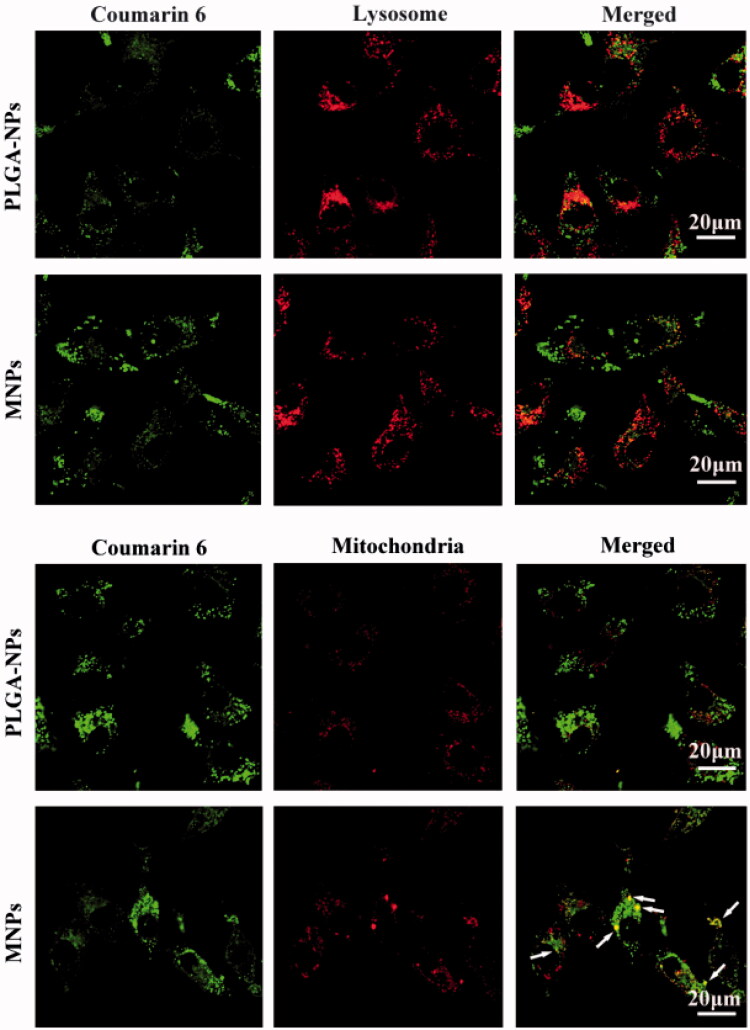
Images of intracellular delivery of C6 labeled PLGA-NPs and MNPs in A549 cells. Merged image of the NPs with the dyes was indicated by arrows.

The intracellular fate of internalized NPs was tracked by C6 labeled NPs using confocal laser scanning microscopy (CLSM) and NPs was observed as green fluorescence. As shown in Figure 4, the cells incubated with NPs for 2 h exhibited green fluorescence in the cytoplasm matrix, indicating that NPs were able to be internalized by the cells. When lysosomes were stained by red fluorescence dye, the merged picture of two colors appeared orange, which indicated NPs present in lysosomes. In fact, the most internalized NPs was first placed in the lysosomes and trapped in lysosomes. However, when the mitochondria were stained by red fluorescence, the yellow color in the merged images of MNPs (see white arrows) represented that majority MNPs accumulate in mitochondria. The yellow color did not appear in PLGA-NPs, which confirmed the common NPs could not escape from the lysosomes to mitochondria. Therefore, mitochondria accumulation of MNPs was higher than PLGA-NPs, due to the lysosomes escaping and mitochondria targeting function of MNPs.

### Antitumor mechanism of MNPs *in vitro*

With the hierarchical targeting function described as above, MNPs were expected to have better antitumor effect in lung cancer. The cytotoxicity of yuanhuacine, yuanhuacine/PLGA-NPs, and yuanhuacine/MNPs against A549 cells was quantitatively assessed by MTT assay (Figure S9). At drug concentrations ranging from 0.25 to 20 μmol/L, all the groups had an increasing trend on inhibition rates depending on concentration. However, inhibition activity of yuanhuacine/PLGA-NPs and yuanhuacine/MNPs was more sensitive at low drug concentrations from 0.25 to 10 μmol/L, and the concentration-dependent increase was remarkable, while at high concentrations (20 μmol/L) nearly no enhancement was seen. In special, the yuanhuacine group had higher inhibition than the corresponding NPs groups at 20 μmol/L, which may be affected by incomplete release of drug from NPs. The IC_50_ values for yuanhuacine, yuanhuacine/PLGA-NPs, and yuanhuacine/MNPs were 7.31, 8.82, and 6.44 μmol/L, respectively. These results indicated MNPs could delivery more drugs into cells, which may be brought by the multifunction of yuanhuacine/MNPs, such as ligand binding internalized, endolysosomal escape, and mitochondrial targeting.

**Figure 5.**
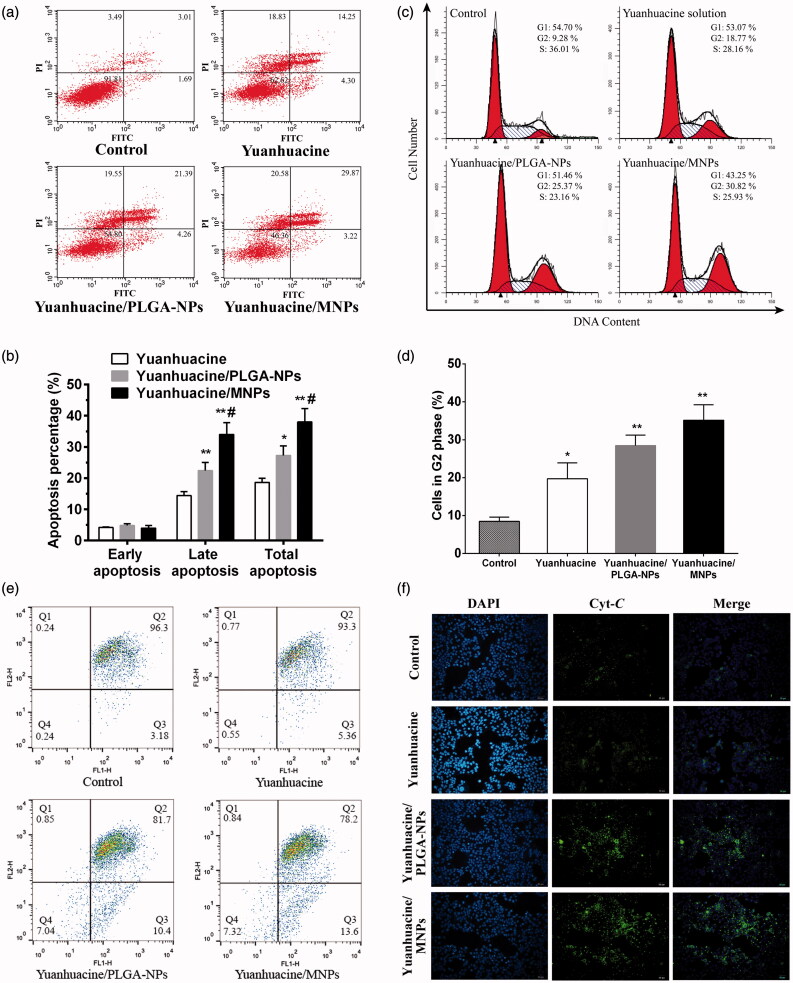
(a) Flow cytometric images to detect apoptosis and (b) apoptosis percentage on A549 cells after applying yuanhuacine, yuanhuacine/PLGA-NPs, and yuanhuacine/MNPs with yuanhuacine concentration of 10 μM for 24 h. *p < .05, **p < .01 versus yuanhuacine, #*p* < .05 versus yuanhuacine/PLGA-NPs. (c) Cell cycle and (d) cell percentage in G2 phase on A549 cells after applying yuanhuacine, yuanhuacine/PLGA-NPs, and yuanhuacine/MNPs with yuanhuacine concentration of 10 μM for 24 h. *p < .05, **p < .01 versus control. (e) Mitochondrial membrane potential measurement and (f) cytochrome c detection on A549 cells after applying yuanhuacine, yuanhuacine/PLGA-NPs and yuanhuacine/MNPs at a yuanhuacin concentration of 10 μM for 24 h.

To reveal the mechanism of cell proliferation inhibition based on the MTT results, so we studied the apoptosis effect of yuanhuacine, yuanhuacine/PLGA-NPs, and yuanhuacine/MNPs against A549 cells. The scatter diagram (Figure 5(a)) in bivariate flow cytometry combined with Annexin V-FITC/PI staining could detect normal cells (the lower left quadrant), early apoptotic cells (the lower right quadrant), late apoptotic cells (the upper right quadrant), and necrotic cells (the upper left quadrant) (Chen et al., 2008). The total apoptotic rate was equal to the sum of early and late apoptosis (Figure 5(b)). We can see the apoptosis rates of yuanhuacine, yuanhuacine/PLGA-NPs, and yuanhuacine/MNPs were increasing in turn, 18.55%, 25.65%, and 33.09%, respectively, while the control group was only 4.70% (the data was not shown). Compared with yuanhuacine group, NPs groups had the promoting apoptosis effect. The MNPs were superior in triggering apoptosis compared to the untargeted analog (PLGA-NPs). And the strongest apoptosis effect of yuanhuacine/MNPs may attribute to escape from the lysosome and target to mitochondria of MNPs, which make high drug concentration in the mitochondria. In addition to cell apoptosis effect, cell cycle regulation was a key pathway for cell proliferation (Li et al., 2009). We further studied the A549 cell cycle using flow cytometry technology combined with propidium iodide (PI) staining. The results showed that yuanhuacine had the effect to block the cell cycle on A549 cells, and mainly blocked in the G2/M phase (Figure 5(c)). The retardation effect of G2/M phase was yuanhuacine/MNPs > yuanhuacine/PLGA-NPs > yuanhuacine (Figure 5(d)). These results were corresponding to the cell morphological observation (Figure S10). In the cell apoptosis process, the cell morphological underwent obvious change, including the cell membrane shrinkage, cytoplasmic condensation, endoplasmic reticulum expansion, and nuclear fragmentation into massive or crescent shaped, which fused into multiple apoptotic bodies. From the nuclei staining images, it can be seen that compared to the control cells, a few cell nucleus in the yuanhuacine group shrink into a crescent, while the most remained round and plump with no atrophy. On the yuanhuacine/PLGA-NPs and yuanhuacine/MNPs condition, the nucleus morphological changes are more obvious, appearing more crescent shaped body accompanied by apoptotic bodies. Moreover, yuanhuacine/MNPs seemed to be more apoptotic bodies than yuanhuacine/PLGA-NPs.

Mitochondria played a very important role in the process of cell apoptosis. The drop of the mitochondrial membrane potential (Δ*Ψ_m_*) was considered to be the earliest signal in the process of cascade apoptosis (Ly et al., 2003). Once Δ*Ψ_m_* collapsed, cell apoptosis was irreversible. Fluorescence probe JC-1 was used in Δ*Ψ_m_* measurement, and its state and fluorescence connected tightly with the function of mitochondria (Binet et al., 2014). When the membrane potential of mitochondria was high, JC-1 gathered in the mitochondrial matrix and formed a polymer that it can produce red fluorescence. While the membrane potential was low, JC-1 was a form of solitary and produced green fluorescence. Hence, red and green fluorescence ratio is commonly used to measure the percentage of mitochondrial depolarization, and it is easy to detect cell membrane potential. In Figure 5(e), the fluorescence images of yuanhuacine/PLGA-NPs and yuanhuacine/MNPs were obviously different from that of control, indicating their Δ*Ψ_m_* was significantly changed with the occurrence of apoptosis. Fall in mitochondrial membrane potential enhanced mitochondrial membrane permeability, subsequently induced the release of cytochrome c (Cyt *C*) from mitochondria into cytoplasm. Cyt *C* is an important component of the mitochondrial respiratory chain, which could induce activation of caspase, leading to cell death. This study use the method of immunofluorescent staining to inspect the release of Cyt *C*. In Figure 5(f), we can see that Cyt *C* was seem to be not detected in control cells, while yuanhuacine, yuanhuacine/PLGA-NPs, and yuanhuacine/MNPs had an increasing Cyt *C* amount indicating increasing apoptosis cell. The enhancement of mitochondrial Cyt *C* release was associated with loss of mitochondrial membrane potential, and both results proved apoptosis pathway was placed in mitochondrial. So mitochondrial targeted drug delivery was conducive to stimulate mitochondrial-related apoptosis pathway.

## Conclusions

In summary, our work was the first report to use hierarchical target strategy for lung cancer drug delivery *via* inhaled administration. Inhaled administration was convenient for first-stage target to the lung tissue of drug carriers, and the RGDfk ligands on surface of nanoparticles improved second-stage targeting to the cancer cell, then intracellular proton sponge effect triggered by the positive charge of histidine in acidic condition achieved the third-stage targeting to mitochondria. These stages were sequentially triggered and aimed at precise drug delivery. The smart MNPs were given hierarchical target function to realize mitochondria-level drug delivery and to increase the mitochondria-related apoptosis, which brought the enhanced efficiency of chemotherapy drugs. This report provides a valid tactic for effective and precise pulmonary drug delivery.

## Supplementary Material

IDRD_Chen_et_al_Supplemental_Content.docx
