# Supplementary material for: Hierarchical pulmonary target nanoparticles via inhaled administration for anticancer drug delivery
Source: Drug Deliv. 2017 Aug 28;24(1):1191–203. doi: 10.1080/10717544.2017.1365395 (PMC8241141; doi:10.1080/10717544.2017.1365395)
Supplement: IDRD_Chen_et_al_Supplemental_Content.docx [file IDRD_A_1365395_SM6785.docx]

Supporting Information


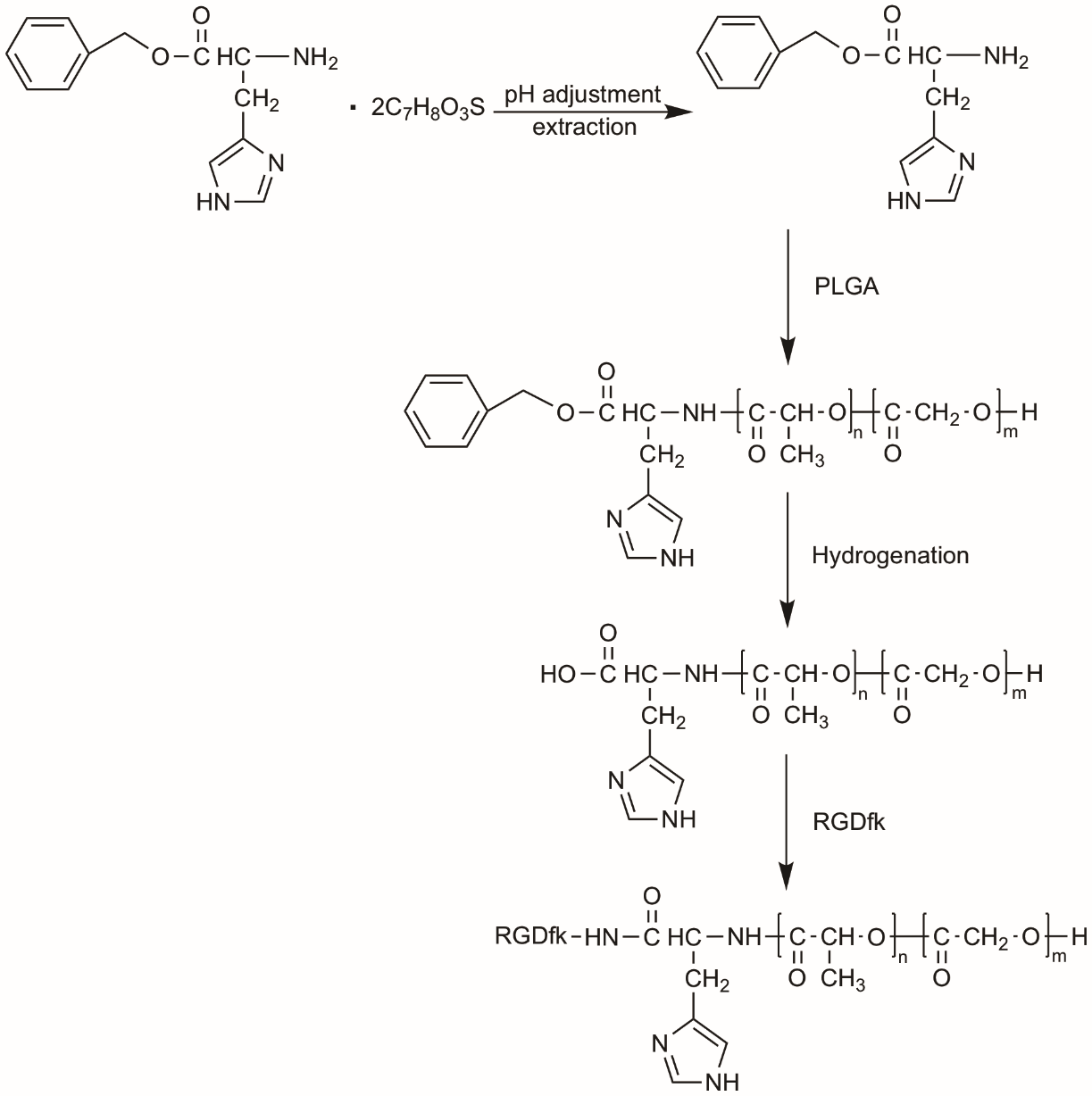


Figure S1. The synthesis of RGDfk-histidine-PLGA included four steps chemical reaction. Step1 histidine benzyl ester was obtained by extraction; Step2 the histidine benzyl ester-PLGA was prepared by amide condensation reaction between amino group on histidine benzyl ester and carboxyl groups on PLGA; Step3 the histidine benzyl ester-PLGA was hydrogenated to obtain histidine-PLGA; Step4 the synthesis of RGDfk-histidine-PLGA involved amide condensation reaction between histidine-PLGA and RGDfk.


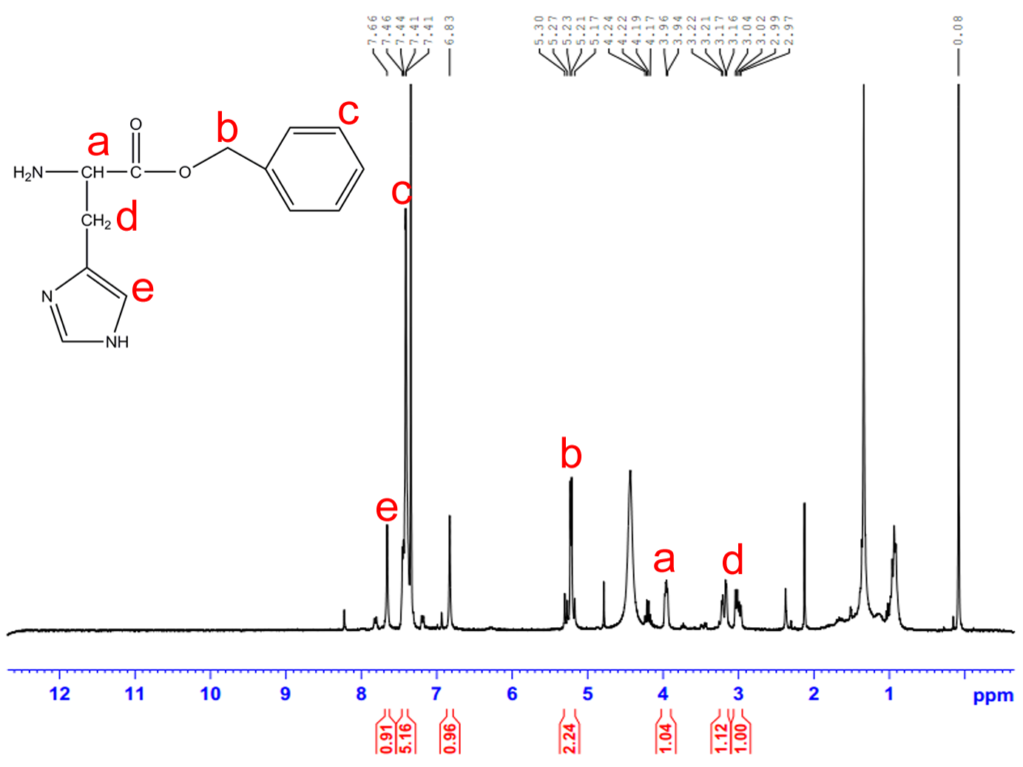


Figure S2. ^1^H NMR spectra of histidine benzyl ester (400 MHz, CDCl_3_): δ=3.96 ppm[s, H-a], δ=5.34 ppm [m, H-b]，δ=7.46 ppm [m, H-c], δ=3.16 ppm [m, H-d], δ=7.66 ppm [s, H-e], H-d：H-a = 2:1 was in line with histidine formula.


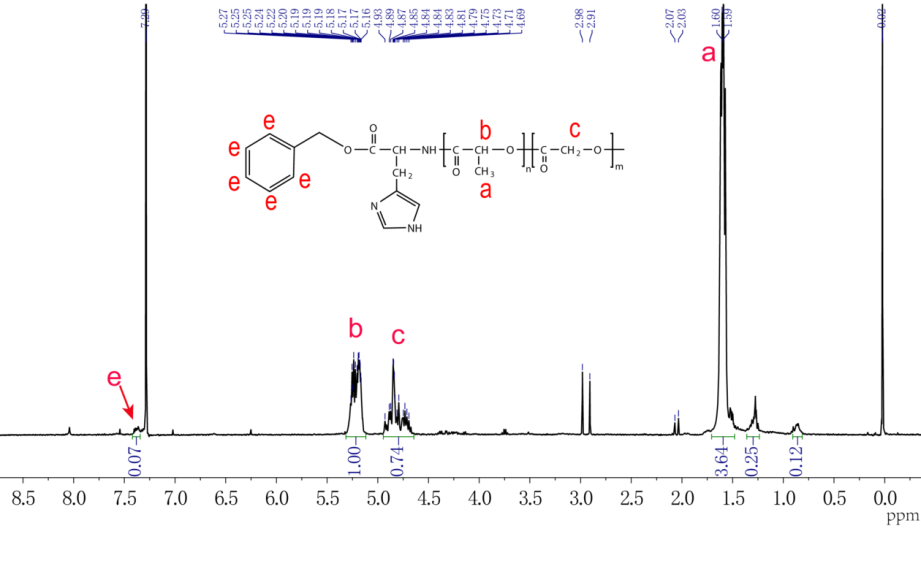


Figure S3. ^1^H NMR spectra of histidine benzyl ester-PLGA (400 MHz, CDCl_3_): δ=1.60 ppm[m, H-a], δ=5.25 ppm [m, H-b]，δ=4.82 ppm [m, H-c]，δ=7.32 ppm [m, H-e] and H-b：H-c =1:0.74 was consistent with the proportion of lactic acid and glycolic acid in the PLGA.


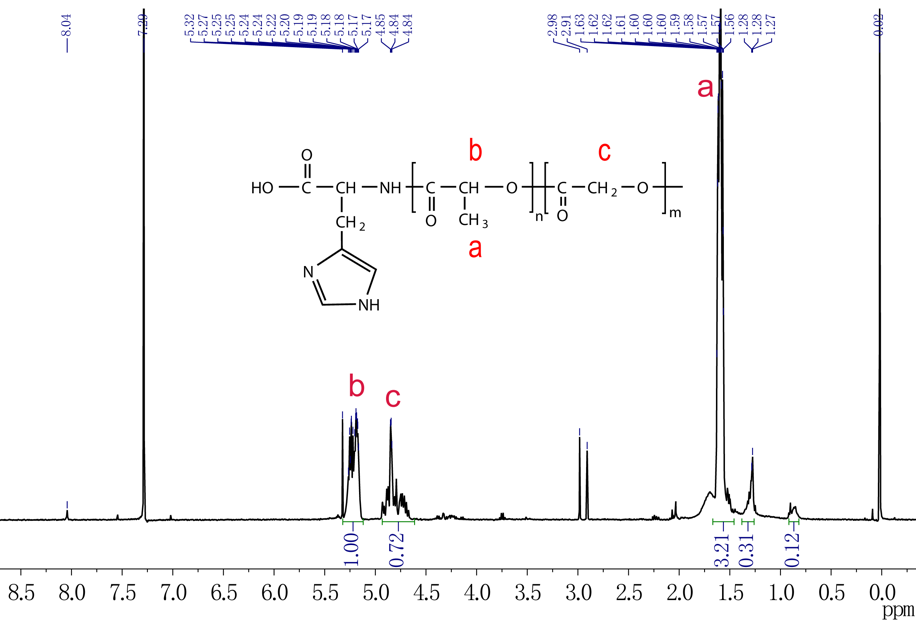


Figure S4. ^1^H NMR spectra of histidine-PLGA (400 MHz, CDCl_3_): δ=1.60 ppm[m, H-a], δ=5.25 ppm [m, H-b], δ=4.82 ppm [m, H-c] and H-b：H-c =1:0.74 was consistent with the proportion of lactic acid and glycolic acid in the PLGA. The disappearance of H-e indicated the success of removed benzyl by hydrogenation .

^
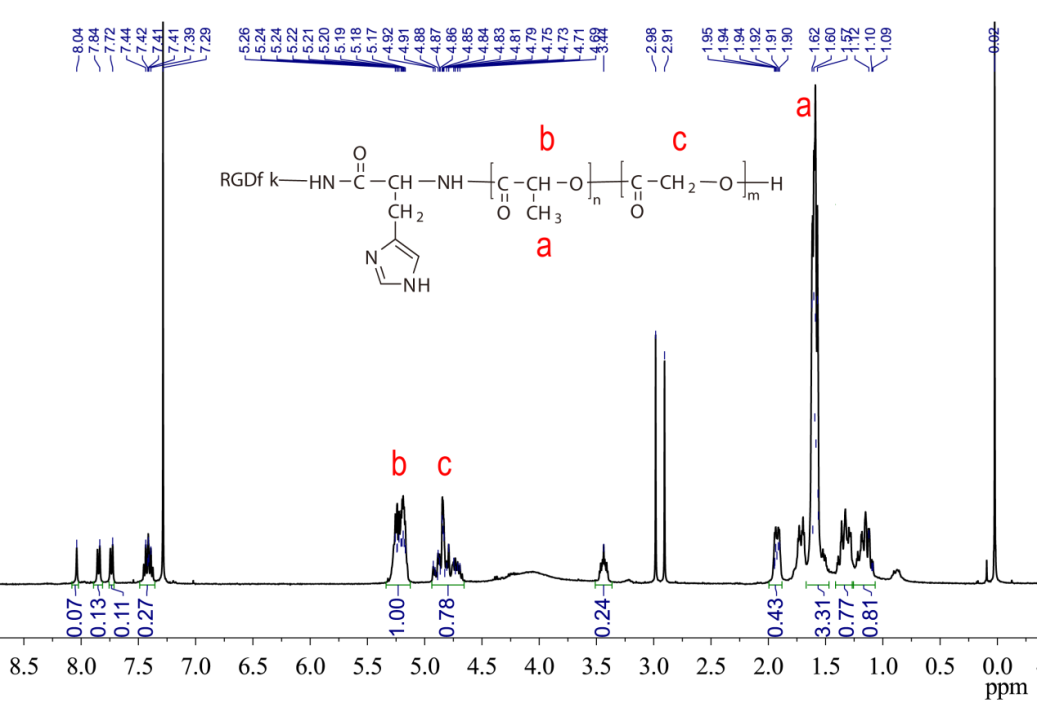
^

Figure. S5 ^1^H NMR spectra of RGDfk-histidine-PLGA (400 MHz, CDCl_3_): δ=1.60 ppm[m, H-a], δ=5.25 ppm [m, H-b]，δ=4.82 ppm [m, H-c] and H-b：H-c =1:0.74 was consistent with the proportion of lactic acid and glycolic acid in the PLGA. Many new small peaks in the range of δ=7.14-8.04 was hydrogens on the benzene of RGDfk.





Figure S6. Zeta potential change of PLGA-NPs and MNPs in pH 3,5 and 7.

Table S1. Size distribution, encapsulation efficiency and loading content of yuanhuacine/NPs

| Yuanhuacine /NPs | Size (nm) | PDI | EE (%) | LC (%) |
| --- | --- | --- | --- | --- |
| PLGA-NPs | 120.3 ± 7.4 | 0.174 | 87.6±4.67 | 4.1 ± 0.13 |
| MNPs | 153.4 ± 6.9 | 0.130 | 90.5±2.42 | 4.3 ± 0.12 |

PDI= polydispersity index, EE= encapsulation efficiency, LC= loading content; Data represented as mean ± standard deviation (n=3).





Figure S7. *In vitro* yuanhuacine release from PLGA-NPs and MNPs.


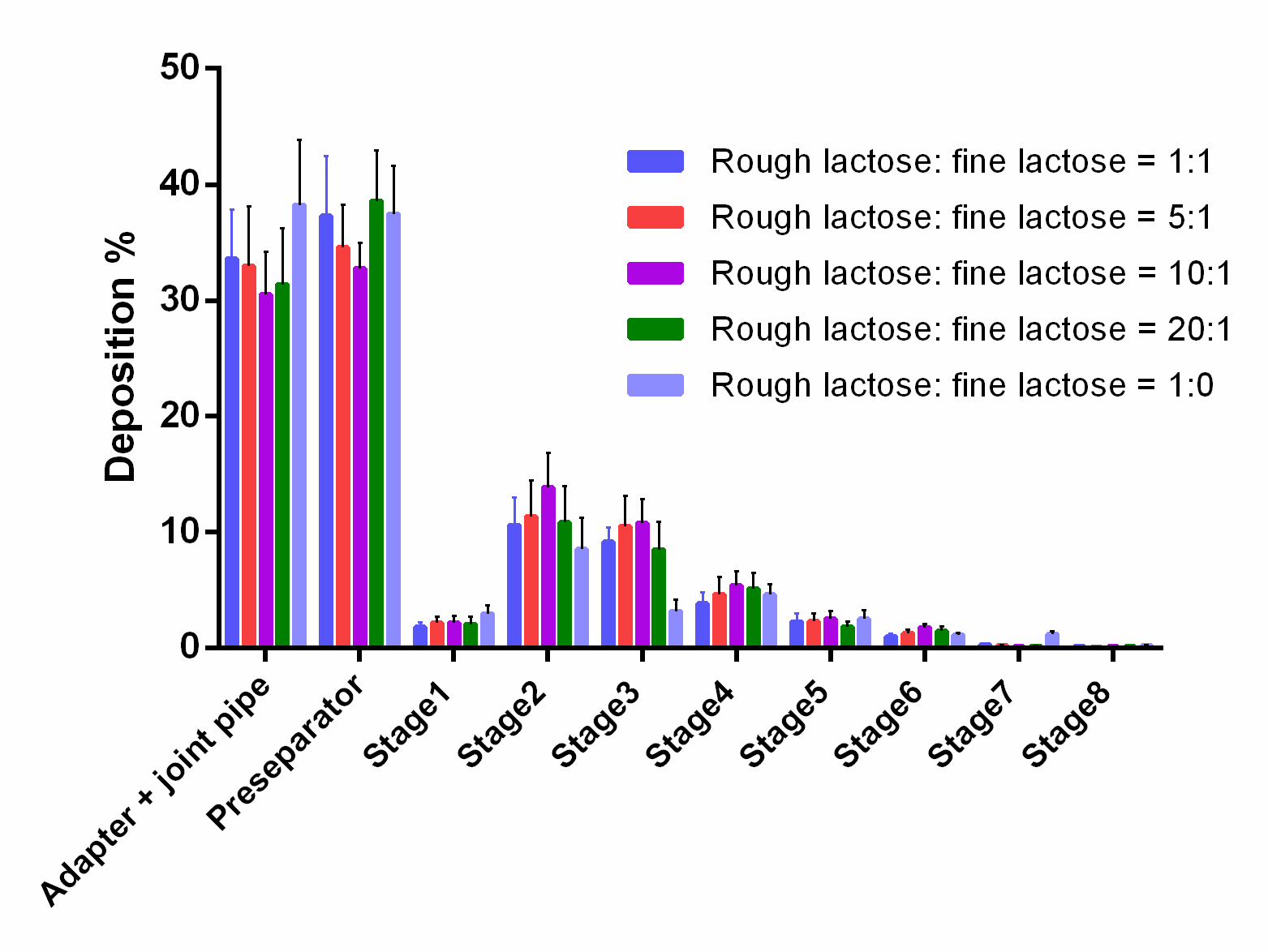


Figure S8. Detailed deposition of yuanhuacine dry power with different prescription in NGI device (n=3). The particle size of each layer: Stage1 (>8.06μm), Stage2 (4.46-8.06 μm), Stage3 (2.82-4.46 μm), Stage4 (1.66-2.82 μm), Stage5 (0.94-1.66 μm), Stage6 (0.55-0.94 μm), Stage7 (0.34-0.55 μm), Stage8 (<0.34 μm)


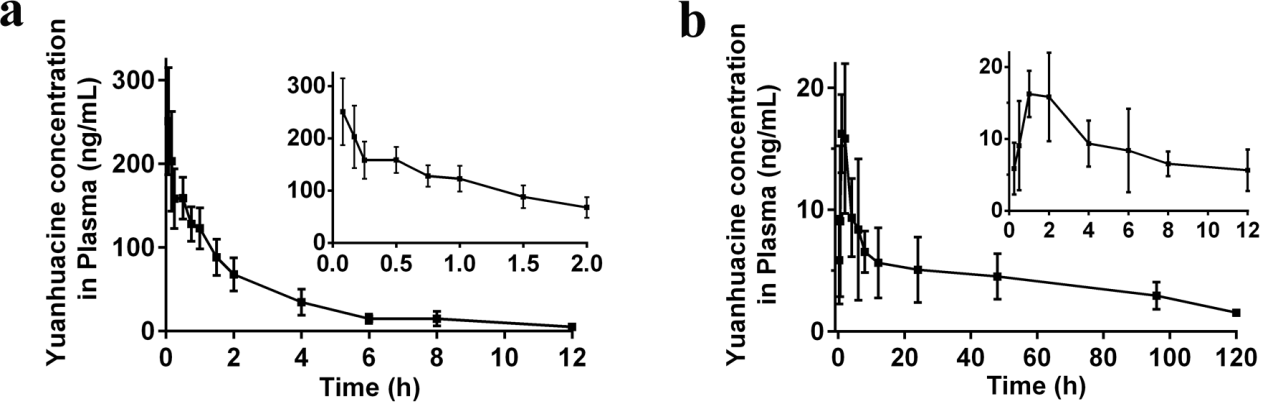


Figure S9. Plasma concentrations of yuanhuacine vs time curves after (a) intravenous and (b) inhaled administration of yuanhuacine /MNPs at a yuanhuacine dose of 100 μg/kg.

Table S2. Pharmacokinetic parameters of intravenous or inhaled yuanhuacine/MNPs in rats at a dose of 100 μg/kg (*n*=6)

| Parameters | Intravenous | Inhaled |
| --- | --- | --- |
| AUC_0 – t_ (ng/mL•h) | 476.75 ± 189.67 | 519.64 ± 215.50 |
| AUC _0 −∞_ (ng/mL•h) | 495.99 ± 209.20 | 841.51± 419.71 |
| MRT_0− t_ (h) | 2.62 ± 0.41 | 44.97 ± 8.52 |
| MRT_0−∞_ (h) | 3.15 ± 1.77 | 88.456 ± 30.43 |
| t_1/2_ (h) | 2.65 ± 1.06 | 90.88 ±53.02 |
| C_max_ (ng/mL) | 250.96 ± 72.02 | 16.23 ± 4.11 |
| T_max_ (h) | 0.08 | 1.00 |





Figure S10. In vitro cytotoxicity assay of various amount of yuanhuacine, yuanhuacine/PLGA-NPs and yuanhuacine/MNPs against A549 cells after 24 h incubation. Data are presented as the mean ± standard deviation (n = 6).

## **
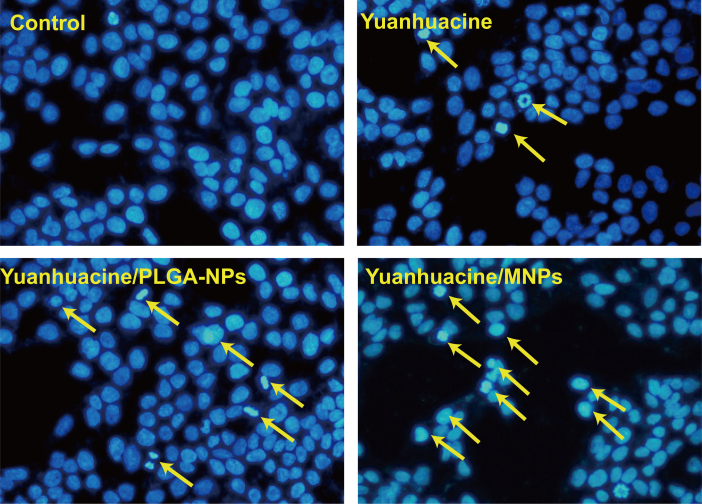
**

## Figure S11. Fluorescent images of apoptosis induction on A549 cells for nuclei shrinking and noticeable nuclear condensation (signified by yellow arrows) after applying yuanhuacine solution, yuanhuacine/PLGA-NPs and yuanhuacine/MNPs.
